# Bioactive compounds, antioxidant and antimicrobial activities of *Arum maculatum* leaves extracts as affected by various solvents and extraction methods

**DOI:** 10.1002/fsn3.815

**Published:** 2019-01-28

**Authors:** Reza Farahmandfar, Reza Esmaeilzadeh Kenari, Maryam Asnaashari, Dina Shahrampour, Tahmineh Bakhshandeh

**Affiliations:** ^1^ Department of Food Science and Technology Sari Agricultural Sciences & Natural Resources University (SANRU) Sari Iran; ^2^ Department of Food Science and Technology Gorgan University of Agricultural Sciences & Natural Resources Gorgan Iran

**Keywords:** Antimicrobial activity, Antioxidant activity, *Arum maculatum*, Solvent extraction, Ultrasound

## Abstract

The different species of *Arum maculatum* plant can be found in all over the world, and a wide range of medicinal applications has been mentioned for them. Thus, it can also be valued as a source of natural compounds with antioxidant and antimicrobial activities. In this study, the effect of solvents (water, ethanol, ethanol:water (50:50)) and extraction methods (maceration and ultrasound) on the extraction yields and bioactive properties of extracts were analyzed. The antioxidant capacity of *Arum maculatum* leaves extracts was investigated, and the concentrations of total phenolics, tocopherols, tannins and flavonoids were determined. 1,1‐diphenyl 2‐picrylhydrazyl free radical (DPPH), β‐Carotene bleaching, and oxidative stability index (OSI) were used to determine antioxidant activity. The ability to scavenge radicals was measured in these experiments by the discoloration of the solution. Also, the antimicrobial activity of different extracts against Gram‐positive (*Staphylococcus aureus and Listeria monocytogenes*) and Gram‐negative bacteria (*Escherichia coli, Salmonella enteritidis,* and *Pseudomonas aeruginosa)* was evaluated by using of microdilution and agar diffusion assays. The results demonstrated that ultrasonic extracts (especially ethanol:water (50:50) solvent) had the higher extraction yield and antioxidant potential than maceration extracts. All extracts were effective against all tested bacteria, and *Listeria monocytogenes* was the most sensitive bacterium with lowest MIC value (12.5 mg/ml) and biggest diameter of growth inhibition zone (13.77 mm). Generally, this *Arum maculatum* leaves extracts can be suggested as an economical source of antioxidant and antimicrobial agents and can be a suitable substitute for artificial and chemical food preservatives.

## 1 INTRODUCTION

Lipid oxidation is one of the serious hazards in food industry that causes off‐flavors and reducing the nutritional value and generates oxidized primary and secondary products. For this reason, synthetic antioxidants which have been used in food products for more than five decades have various adverse health effects (Eshghi, Asnaashari, Haddad Khodaparast, & Hosseini, 2014; Farahmandfar, Asnaashari, & Sayyad, 2015). Natural antioxidants are an interesting concept for scientists which could find good alternative for synthetic antioxidants (Asnaashari, Tajik, & Khodaparast, 2015). Plants and herbs were introduced as a potential source of natural antioxidants in recent years (Farahmandfar et al., 2015). Bioactive compounds of plants and herbs like flavonoids, tannins, and hydroxycinnamate esters can provide antioxidant properties and beneficial effects like antimutagenic, anticarcinogenic, and cardioprotective activities (Asnaashari, Hashemi, Mohammad, Mehr, & Asadi Yousefabad, 2015; Farhoosh, Johnny, Asnaashari, Molaahmadibahraseman, & Sharif, 2016). Primary antioxidants directly scavenge free radicals, while secondary antioxidants indirectly inhibit the generation of free radicals through Fenton's reaction (Oh, Jo, Cho, Kim, & Han, 2013). The researchers also revealed the antioxidant capacity of phenolic compounds occurs mostly through a redox mechanism and allows the compounds to act as hydrogen donors, reducing agents, metal chelators, and singlet oxygen quenchers and prevent the formation of free radicals (Asnaashari, Asnaashari, Ehtiati, & Farahmandfar, 2015).

Actually, oxidative stress condition occurs with the steady increase of free radicals in cells that causes oxidize blood vessel walls, protein molecules, lipids, and DNA which can result in creating cancerous cells and different diseases (Hashemi, Khaneghah, Tavakolpour, Asnaashari, & Mehr, 2015). Researchers have reported that these harmful effects can be reduced by regular consumption of foods and beverages which exhibit antioxidant activity (Farhoosh, Sharif, Asnaashari, Johnny, & Molaahmadibahraseman, 2016). Moreover, medicinal plants play a crucial role to cure basic health problems (Asnaashari, Farhoosh, & Farahmandfar, 2016). So, try to achieve new natural antioxidative and antimicrobial compounds due to a people tendency to consumption of food without chemical preservatives has been increasing. Herbal extracts have shown potential antioxidant potency and antimicrobial activity against different organisms in various studies (Almajano, Carbo, Jiménez, & Gordon, 2008; Oh et al., 2013).

In this investigation, *Arum maculatum* plant was selected to assess its antioxidant and antibacterial properties. This plant belongs to *Arum family* (*Araceae*) which numbers nearly 1,000 members, mostly tropical, and many of them are marsh or water plants. There is an only one species of these plants in Iran and known as “cardin” that the local people use the leaves of it as a vegetable (Al‐Qura'n, 2005).

A wide range of medicinal applications has been mentioned for *Arum maculatum* species; for example, the roots and fresh plants are used in such treatments diaphoretics, expectorants, vermifuges, and rheumatic pain (Nabeel, Abderrahman, & Papini, 2008). The phytochemical screening of *Arum* plants showed that these plants contain alkaloids, polyphenols, glycosides (flavonoids, saponin, and cyanogenic groups), proanthocyanidins, 2‐heptanone, indoles, p‐cresol, (E)‐caryophyllene, monoterpenes, two sesquiterpenes, and lectin (Safari, Amiri, Bahador, Amiri, & Esmaeili, 2014). Therefore, it is necessary to select an efficient method for extraction of these vital components. Different novel extraction techniques have been developed for the extraction of antioxidative compounds from plant sources. Among the non‐conventional methods, ultrasonic‐assisted extraction (UAE) technique can offer with high reproducibility in short times, simple manipulation, reduced solvent consumption and temperature, and lower energy input (Farahmandfar et al., 2015; Farahmandfar, Asnaashari, & Sayyad, 2017).

As far as we know, there are few studies about the biological activity of *Arum maculatum* species. For example, Dayisoylu (2010) investigated the antioxidant activity of this plant leaves from various locations in a province of Turkey by measuring antioxidant enzymes such as superoxide dismutase (SOD) and catalase (CAT).

Moreover, biological effects of ethanolic and methanolic extracts of *Arum maculatum* compared with another plant were assayed (Safari et al., 2014). Also in another study antioxidant and antimicrobial activities of essential oil of this plant were determined (Kianinia & Farjam, 2018).

Thus, the objectives of this study were (a) to extract total phenolic compounds, tannin, flavonoid, and tocopherol from Iranian specie of *Arum maculatum* with ultrasound and maceration methods, (b) to compare extraction yield in different methods with various solvents, (c) to investigate the antioxidative effects of *Arum maculatum* through the use of several accepted in vitro assays: DPPH radical scavenging activity, OSI (Rancimat test) and Beta‐Carotene/linoleic bleaching inhibition, and (d) to evaluate the antibacterial properties of *Arum maculatum* extracts.

## 2 MATERIALS AND METHODS

### 2.1 Chemicals and reagents

All reagents and solvents used in the analysis are analytical grade, and microbial cultures (like nutrient broth, nutrient agar, and Mueller Hinton agar) were obtained from Merck (Darmstad, Germany) and Sigma Aldrich (St. Louis, MO, USA) companies. Butylated hydroxyanisole (BHA) used as a standard antioxidant was provided from TITRAN, and Blank paper and Gentamicin disk (10 μg per disc) were provided from Padtan Teb Company.

### 2.2 Preparation of *Arum maculatum* plant extract

In this research, fresh leaves of *Arum maculatum* were randomly collected from the fields of Shiraz in the Fars, Iran in 2016. The leaves of *Arum maculatum* were separated, cleaned, and dried under shade at room temperature for 1 week. Dried leaves were powdered mechanically by a mixer (Panasonic, MK‐G20NR).

### 2.3 Extraction procedures

#### 2.3.1 Maceration

Ten grams of the powdered *Arum maculatum* leaves was separately extracted by water, ethanol, and mixture of ethanol:water (50:50) in a shaker for 12 hr in 160 rpm at room temperature. After filtering twice through the Whatman No.1 filter paper and removing the solvents by vacuum oven, the residual crude water, ethanolic and ethanol:water (50:50) of *Arum maculatum* leaves extracts were weighted and stored at −18°C (Farahmandfar et al., 2015).

#### 2.3.2 Ultrasound‐assisted extraction (UAE)

The mixture of *Arum maculatum* powdered leaves with water, ethanol, and ethanol:water (50:50) was sonicated for 30 min in an ultrasonic bath (Elmas 30 H model, operating at 20 kHz frequency) at 45°. The extracts were filtered, and the further steps were done like previous section (Sayyari & Farahmandfar, 2017).

### 2.4 Chemical properties of extracts

#### 2.4.1 Total phenolic content

Total phenolic content of the *Arum maculatum* extracts was determined by the method described by Farahmandfar et al. (2015). At first, 0.5 ml of the extracts were mixed with Folin–Ciocalteu reagent (2.5 ml). Then, sodium carbonate solution (7.5%, 2 ml) adjusted to 50 ml water. And finally, the absorbance of the *Arum maculatum* samples was read at 765 nm. The phenolic content was expressed as mg gallic acid equivalents per gram of the extract.

#### 2.4.2 Total tocopherol content

The tocopherol content was determined spectrophotometrically at 520 nm based on Farahmandfar and coworkers, 2015 method (Farahmandfar et al., 2015). The *Arum maculatum* sample was weighed and toluene (C_7_H_8_) (5 ml), 2, 2’‐bipyridine (0.07% w/v in 95% aqueous ethanol, 3.5 ml), and FeCl_3_.6H_2_O (0.2% w/v in 95% aqueous ethanol, 0.5 ml) were added. Then, the solution was made up to 10 ml with 95% aqueous ethanol. After one min, the absorption was measured.

#### 2.4.3 Total tannin content

Tannins are water soluble polymers whose monomer units are phenols. Therefore, some steps of tannin determination are similar to those of phenols. Total tannin content was determined by the Folin–Ciocalteu procedure, as Blainski, Lopes, and De Mello (2013) method. After their adsorption onto hiding powder the polyvinylpolypyrrolidone (PVPP), samples of *Arum maculatum* leaves extract were homogenized with adsorbent material and stirred for one hour and then stored for at 4°C for 2 hr to develop tannin–PVPP complex at pH = 3. After centrifugation at 1,792 *g* for 15 min, no adsorbed phenolics in the supernatant were determined by the Folin–Ciocalteu. The calculated total tannins of the *Arum maculatum* extract were subtracted from total polyphenol contents and expressed as mg gallic acid equivalents per gram (mg gallic acid/g).

#### 2.4.4 Total flavonoid content

25 μL of aqueous, ethanolic, and ethanol:water (50:50) extracts of *Arum maculatum* leaves extract was individually added to 125 μl of H_2_O. Afterward, NaNO_2_ (5%, 7.5 μl), AlCl_3_ (10%, 15 μl), and NaOH (1 M, 50 μl) were added to the mixture. The mixture was diluted by adding H_2_O (27.5 μl), and the absorbance was read at 510 nm against a blank. The results were expressed as mg catechin equivalents per gram (mg CE/g).

### 2.5 Antioxidant activity

#### 2.5.1 DPPH assay

The antioxidant activity was measured by radical scavenging ability of 1, 1‐diphenyl‐2‐picrylhydrazyl (DPPH) radical. The free radical scavenging capacity of aqueous, ethanolic, and ethanol:water (50:50) extracts of *Arum maculatum* leaves was measured using the method described by Asnaashari, Farhoosh, and Sharif (2014) with some modifications. The *Arum maculatum* samples were added into tubes containing 5.9 ml of 0.1 mM methanolic DPPH solution. The reaction mixtures were shaken and kept in the dark. Finally, the decrease in absorbance at 517 nm was measured. The results were calculated using the following equation.

(1) $$\%\;\text{Radical scavenging capacity}=\left(A_{B}-A_{A}\right)/A_{B}*100$$

where:

A_B_ = absorption of blank sample

A_A_ = absorption of sample solutions

#### 2.5.2 Beta‐Carotene/linoleic bleaching inhibition

β‐carotene (0.1 mg) was added to a flask with linoleic acid (20 mg) and Tween 40 (200 mg); all dissolved in chloroform. After evaporation to dryness under vacuum at 50°C distilled water (50 ml), saturated with gas was slowly added. The aqueous, ethanolic, and ethanol:water (50:50) extracts of *Arum maculatum* leaves were solubilized in methanol with HCl (0.01%), and 0.2 ml of the mixture was added to 5 ml of the beta‐carotene/linoleic acid emulsion in order to obtain the final concentration of 0.2 mg ml^−1^. The control sample contained 0.2 ml water and 5 ml β‐carotene/linoleic acid emulsion. Immediately after, the emulsion was added to each tube, the zero time absorbance was read at 470 nm, and then, the samples were incubated in a water bath at 50°C for 60 min. The results were calculated as the percent inhibition using the following equation:

(2) $$\%\text{AA}=\left(DR_{c}-DR_{s}\right)/DR_{c}*100$$

where:

AA: antioxidant activity

DR_c_: degradation rate of control

DR_s_: degradation rate of sample

#### 2.5.3 Oxidative Stability Index (OSI)

A good method to determine the vegetable oil stability of vegetable oils is the oxidative stability index (OSI), which is obtained by Rancimat. In this acceleration method, air is passed through the oil samples, which helps in the rapid oxidation of the triglycerides. For this purpose, 100 ppm of the aqueous, ethanolic, and ethanol:water (50:50) (maceration and ultrasound treatments) of *Arum maculatum* leaves extract and 100 ppm of BHA (as control antioxidant) were exposed to Rancimat (Metrohm model 734, Herisan Switzerland) at 120°C at an airflow of 15 L/hr. Measuring equipments were cleaned three times before the experiments.

### 2.6 Antimicrobial activity

#### 2.6.1 Bacterial strains

Two strains of Gram‐positive bacteria (*Staphylococcus aureus and Listeria monocytogenes*) and three strains of Gram‐negative bacteria (*Escherichia coli, Salmonella enteritidis,* and *Pseudomonas aeruginosa*) as indicator microorganisms were used to evaluate the antibacterial activity of *Arum maculatum* leaves extracts. All bacterial strains were obtained from Iranian Research Organization for Science and Technology (IROST). Each bacterium was subcultured in 10 ml nutrient broth (24 hr, 37°C) and kept frozen at −20°C in broth media supplemented with 25% (v/v) glycerol as a stock culture. Before the antimicrobial assays, they were inoculated into the nutrient broth and incubated at 37°C for 24 hr.

#### 2.6.2 Microdilution assay

Minimum inhibitory concentrations (MIC) and minimum bactericidal concentrations (MBC) of the *Arum maculatum* extracts were determined by using a standard microdilution technique in nutrient broth tubes. In each test batch, a total of 12 glass tubes were used, in which three tubes were considered as controls. These included a negative control (tube containing extract and growth medium without inoculum and a positive control (tube containing growth medium inoculum with bacteria). In test tubes, 500 μl of every extracts with different concentration (12.5–100 mg/ml) was added to 10 ml nutrient broth inoculated with overnight culture of indicator bacteria (10^8^ cfu/ml). All tubes were placed for 24 hr at 37°C. MIC values were taken as the lowest concentration of extracts that produced no visible bacterial growth when compared with the control tubes after 24 hr of incubation at 37°C. In order to evaluate MBC, 100 μl of each MIC tubes and next tubes with higher concentration of extracts was placed on nutrient agar and incubated at 37°C for 24 hr. The lowest concentration which no bacterial growth observed on nutrient agar plates was considered as MBC (Almajano et al., 2008).

#### 2.6.3 Disk diffusion assay

This antimicrobial test was performed as described by Nitiema, Savadogo, Simpore, Dianou, and Traore (2012) with some modification. First, each overnight culture of five tested bacteria with 10^8^ cfu/ml concentration was inoculated over the Mueller–Hinton agar plates using sterile swab sticks, and then, six‐mm paper disks were impregnated with 5 μl of different extracts placed aseptically on the agar surface. Gentamicin disks (10 μg) were used as positive control. The plates were incubated at 37°C for 24 hr, and zones of inhibition were subsequently measured by using a ruler.

#### 2.6.4 Agar well diffusion assay

In agar well diffusion assay, a suspension of each bacterium in physiological serum adjusted to 0.5 McFarland turbidity standard with 10^8^ cfu/ml concentration and was seeded on the surface of plates containing Mueller–Hinton agar (MHA). Afterward wells of 6 mm in diameter were formed and filled with 150 μl of different extracts. These plates were incubated at 37°C for 24 hr. Antimicrobial activity was recorded by measuring the diameter of the inhibition zone around the wells. Antagonistic properties were reported as negative if no inhibition zone was observed (Bubonja‐Sonje, Giacometti, & Abram, 2011).

All the antibacterial assays were performed in triplicate, and the results are expressed as the mean value of the experiments.

### 2.7 Statistical analysis

An analysis of variance (ANOVA) was performed according to SPSS software. Significant differences between means were determined by Duncan's multiple range tests. Differences at *p* < 0.05 were considered to be significant.

## 3 RESULTS AND DISCUSSION

### 3.1 Yield extraction

Table 1 shows the levels of constituents and the yield of extraction by different methods. The bioactive components of the extracts from various solvent extraction methods depend on plant matrix and choosing a suitable solvent of extract (Rezaie, Farhoosh, Iranshahi, Sharif, & Golmohamadzadeh, 2015). Also, solvent viscosity as a physical property can affect on the extractability of bioactive constituents from plant materials. So that low‐viscosity solvents have high diffusivity into the pores of the plant matrices and can leach out the bioactive compounds. The ultrasonic treatment is considerably affected by viscosity and also vapor pressure of various solvents. Ultrasonic treatment in solvents with low‐vapor pressures produces few cavitation bubbles which implode with extensive force, which increase plant matrix disruption. In this research, ethanol:water (50:50) ultrasonic treatment of *Arum maculatum* leaves extract had the highest yield of extraction (32.47%). Results showed that ultrasonication enhanced the yield of *Arum maculatum* extraction compared to maceration (Table 1). Also, type of solvents effect on the extraction yield in both procedures and combination of solvents (ethanol and water) increased diffusibility due to the stronger hydrogen bonds.

**Table 1:** Total phenolic, tannin, flavonoid, tocopherol contents of *Arum maculatum* leaves extracts from different extraction methods

Samples	Total phenolics^1^	Total tannins^1^	Total tocopherols^2^	Total flavonoids^3^	Yield (%)^4^
Water maceration	39.85 ± 0.3^d^	2.96 ± 0.62^b^	112.11 ± 2.2^c^	1.67 ± 0.65^c^	25.63 ± 0.6^c^
Ethanol:water(50:50)maceration	51.09 ± 1.43^b^	5.33 ± 1.4^a^	110.59 ± 6.05^c^	4.17 ± 1.25^a^	26.85 ± 0.91^bc^
Ethanol maceration	46.64 ± 1.48^c^	4.27 ± 0.12^ab^	109.62 ± 6.72^c^	3.45 ± 0.38^ab^	25.69 ± 0.54^c^
Water ultrasonic	41.69 ± 0.99^d^	3.5 ± 0.37^b^	126.38 ± 6.7^ab^	1.96 ± 0.59^c^	27.3 ± 0.99^b^
Ethanol:water(50:50)ultrasonic	55.25 ± 0.96^a^	5.27 ± 0.28^a^	133.48 ± 3.11^a^	4.42 ± 0.73^a^	32.47 ± 0.85^a^
Ethanol ultrasonic	51.18 ± 1.58^b^	3.37 ± 0.72^b^	123.4 ± 3.79^b^	2.65 ± 0.75^bc^	28.25 ± 0.98^b^

Note. Different letters in the column indicate significant differences (*p* < 0.05).

1 *mg* of gallic acid/*gr* sample.

2 μ*g* of α‐ tocopherol/*ml* sample.

3 mg catechin/gr sample.

4 Data expressed as grams of dry extract per 100 g of dried plant material.

### 3.2 Total phenolic content

Phenolic compounds are known as powerful antioxidants due to their ability to scavenge free radicals, singlet oxygen, and superoxide radicals. The radical scavenging activity is attributed to hydroxyl groups replacing in the aromatic ring of the phenolic components. The total phenolic content of *Arum maculatum* leaves extracted by different extraction methods is shown in Table 1. The samples extracted by ethanol:water (50:50) ultrasonic had the highest phenolic content (55.25 mg/g), followed by ethanol ultrasonic (51.18 mg/g) and ethanol:water (50:50) maceration (51.09 mg/g), ethanol maceration (46.64 mg/g), water ultrasonic (41.69 mg/g), and water maceration (39.85 mg/g). Indeed, a direct relationship was found between the total phenolic content of *Arum maculatum* leaves extract and the solvent polarity. Ethanol:water (50:50) mixture and ethanol could remarkably extract higher amounts of phenolic compounds than water. This can be attributed to the fact that alcohols take part in the chemical reactions including carbon–oxygen and oxygen–hydrogen bonds, while water molecules can only participate in oxygen–hydrogen bonds. In alcohols, the unshared electron pair of the oxygen atom are more accessible than that of water molecules (Rezaie et al., 2015).

### 3.3 Total tannin contents

Tannins which have radical scavenging ability can be widely found in plants and have some beneficial effects in health. As shown in Table 1, ethanol:water (50:50) maceration of *Arum maculatum* leaves extract (5.33 mg/g) and ethanol:water (50:50) ultrasonic (5.27 mg/g) had the highest total tannin content, followed by ethanol maceration (4.27 mg/g), water ultrasonic (3.5 mg/g), ethanol ultrasonic (3.37 mg/g), and water maceration (2.96 mg/g).

A comparison of the two groups of tannins and flavonoids in Table 1 demonstrated that tannins have more capacity than flavonoids because almost all the tannins showed considerable scavenging activity in low concentration, whereas the flavonoids activity varied among the different compounds. In fact, increasing the molecular weight, galloyl groups, and ortho‐hydroxyl structure increase the tannin activity, while, hydroxyl groups have important role in flavonoids activity. In addition, when the free hydroxyl group was methoxylated or glycosylated, the radical inhibitory activity was vividly reduced.

### 3.4 Total flavonoid contents

Flavonoids have ability to act as antioxidants in biological systems. The flavonoids antioxidant activity depends on total number and configuration of hydroxyl groups which influence on metal ion chelation capacity, radical scavenging ability, and interaction with enzyme functions. In *Arum maculatum* leaves extraction method, ethanol:water (50:50) ultrasonic (4.42 mg CE/g) and ethanol:water (50:50) maceration (4.17 mg CE/g) had the highest total concentration of flavonoids, followed by ethanol maceration (3.45 mg CE/g), ethanol ultrasonic (2.65 mg CE/g), water ultrasonic (1.96 mg CE/g), and water maceration (1.67 mg CE/g) (Table 1).

It is believed that the dietary intake of flavonoid‐rich foods was suggested due to their ability to donate hydrogen atoms. So, regarding the potentially antioxidant activity of *Arum maculatum* leaves extract, this plant extract can be added to food products to protect them against lipid peroxidation.

### 3.5 Total tocopherol contents

As seen in Table 1, on the basis of total tocopherol content of *Arum maculatum* leaves extracts from various extraction methods, ethanol:water (50:50) ultrasonic with 133.48 μg/ml acted better than other extraction methods including water ultrasonic (126.38 μg/ml), ethanol ultrasonic (123.4 μg/ml), water maceration (112.11 μg/ml), ethanol:water (50:50) maceration (110.59 μg/ml), and ethanol maceration (109.62 μg/ml).

### 3.6 Radical scavenging activity

Free radical chain reaction is widely accepted as the main mechanism of lipid peroxidation (Farhoosh, Johnny, et al., 2016). In order to terminate free radical chain reaction, radical scavenging could react and quench peroxide products. DPPH radical scavenging assay is the most sensitive and simple spectrophotometric methods for antioxidant capacity of plant extract determination. The scavenging effect of *Arum maculatum* leaves extracts was measured in different concentrations (200 to 1,200 ppm) and compared to BHA in 200 ppm. In 200 ppm, radical scavenging activity decreased in the order BHA> ethanol:water (50:50) ultrasonic> ethanol:water (50:50) maceration> water ultrasonic> ethanol ultrasonic~ ethanol maceration~ water maceration and in other concentrations: ethanol:water (50:50) ultrasonic> ethanol:water (50:50) maceration> water ultrasonic> ethanol ultrasonic> ethanol maceration~ water maceration. These results are summarized in Table 2. The present results suggest that the extracts are apparently good free radical scavengers and probably have the ability to inhibit lipid peroxidation.

**Table 2:** Radical scavenging activity of different concentrations of *Arum maculatum* leaves extracts with different extraction methods

Samples	200 ppm	400 ppm	600 ppm	800 ppm	1000 ppm	1200 ppm
Water maceration	20.73 ± 3.05^d^	23.09 ± 3.39^d^	26.93 ± 3.96^d^	35.9 ± 5.28^d^	42.32 ± 6.2^d^	50.48 ± 7.42^c^
Ethanol:water (50:50) maceration	31.19 ± 2.12^bc^	34.73 ± 2.36^ab^	40.52 ± 2.75^ab^	54.00 ± 3.67^ab^	63.6 ± 4.32^ab^	75.9 ± 5.16^ab^
Ethanol maceration	23.4 ± 3.39^d^	26.07 ± 3.77^d^	30.4 ± 4.4^d^	40.53 ± 5.87^d^	47.7 ± 6.92^d^	56.99 ± 8.25^c^
Water ultrasonic	28.37 ± 1.51^c^	31.59 ± 1.68^bc^	36.85 ± 1.96^bc^	49.12 ± 2.61^bc^	57.9 ± 3.08^bc^	69.07 ± 3.68^b^
Ethanol:water (50:50) ultrasonic	34.77 ± 2.93^b^	38.73 ± 3.27^a^	45.17 ± 3.81^a^	60.2 ± 5.08^a^	70.96 ± 5.9^a^	81.39 ± 3.86^a^
Ethanol ultrasonic	24.00 ± 1.6^d^	26.73 ± 1.78^cd^	31.18 ± 2.08^cd^	41.57 ± 2.77^cd^	48.9 ± 3.27^cd^	58.44 ± 3.9^c^
BHA	76.83 ± 1.75^a^					

Note. Different letters in the column indicate significant differences (*p* < 0.05).

### 3.7 Beta‐carotene/linoleic bleaching inhibition

The β‐carotene bleaching assay evaluates the extracts antioxidant activity to protect β‐carotene from the free radicals generated during the linoleic acid peroxidation. In the absence of antioxidant, β‐carotene undergoes rapid discoloration. ß‐carotene/linoleic bleaching inhibition of *Arum maculatum* leaves extract in different concentrations and methods is shown in Table 3. Ethanol:water (50:50) ultrasonic sample had the most inhibition, followed by ethanol:water (50:50) maceration, water ultrasonic, ethanol ultrasonic, ethanol maceration, and water maceration (samples were compared by BHA as a synthetic antioxidant).

**Table 3:** Beta‐carotene/linoleic bleaching inhibition of *Arum maculatum* leaves extracts from different extraction methods

Samples	200 ppm	400 ppm	600 ppm	800 ppm	1000 ppm	1200 ppm
Water maceration	32.41 ± 4.77^d^	47.87 ± 7.04^d^	57.96 ± 8.52^c^	56.57 ± 8.32^c^	58.4 ± 8.59^d^	52.18 ± 7.67^c^
Ethanol:water (50:50) maceration	48.75 ± 3.31^bc^	72.01 ± 4.89^ab^	87.17 ± 5.92^ab^	85.09 ± 5.78^ab^	87.8 ± 5.97^ab^	78.49 ± 5.3^ab^
Ethanol maceration	36.59 ± 5.3^d^	54.04 ± 7.82^d^	65.42 ± 9.47^c^	63.86 ± 9.25^c^	65.94 ± 9.5^d^	58.9 ± 8.53^c^
Water ultrasonic	44.34 ± 2.36^c^	65.5 ± 3.49^bc^	79.29 ± 4.22^b^	77.4 ± 4.12^b^	79.9 ± 4.25^bc^	71.39 ± 3.8^b^
Ethanol:water (50:50) ultrasonic	54.35 ± 4.59^a^	80.2 ± 8.78^a^	93.02 ± 4.42^a^	90.21 ± 4.51^a^	97.9 ± 8.27^a^	84.14 ± 3.99^a^
Ethanol ultrasonic	37.52 ± 2.5^d^	55.42 ± 3.7^cd^	67.09 ± 4.47^c^	65.49 ± 4.37^c^	67.6 ± 4.51 ^cd^	60.41 ± 4.03^c^
BHA	76.83 ± 1.75^a^					

Note. Different letters in the column indicate significant differences (*p* < 0.05).

### 3.8 Oxidative stability index

The OSI is defined as hours needed for the rate of conductivity grows up rapidly to reach a specific content. Longer OSI values demonstrated higher antioxidant activities. Table 4 summarizes oxidative stability index of *Arum maculatum* leaves extracts in different concentrations and extraction methods. The highest stabilizing effect among different extraction methods belonged to ethanol:water (50:50) ultrasonic, followed by ethanol:water (50:50) maceration, ethanol ultrasonic, water ultrasonic, ethanol maceration, and water maceration. The increase of the extract to 1,200 ppm leads to increase in OSI. BHA as a control antioxidant shows higher stabilizing effect in comparison with the *Arum maculatum* leaves extracts in 200, 400, 600, and 800 ppm concentrations but *Arum maculatum* leaves extracts in 1,000 and 1,200 ppm showed upper stability than BHA. So, *Arum maculatum* leaves extract as a natural antioxidant exhibited higher stability than a synthetic one in some concentrations. So that, 1,200 ppm *Arum maculatum* leaves extract could remarkably inhibit lipid oxidation even better than BHA.

**Table 4:** Oxidative stability index of *Arum maculatum* leaves extracts from different extraction methods at 120°C and airflow rate of 15 L/hr

Samples	200 ppm	400 ppm	600 ppm	800 ppm	1000 ppm	1200 ppm
Water maceration	2.78 ± 0.08^d^	2.92 ± 0.08^d^	4.05 ± 0.11^d^	4.57 ± 0.13^cd^	5.1 ± 0.14^cd^	5.26 ± 0.14^cd^
Ethanol:water(50:50)maceration	2.99 ± 0.08^bc^	3.14 ± 0.08^ab^	4.35 ± 0.11^ab^	4.9 ± 0.13^ab^	5.48 ± 0.14^ab^	5.65 ± 0.15^ab^
Ethanol maceration	2.85 ± 0.08^cd^	2.99 ± 0.08^d^	4.14 ± 0.11^d^	4.67 ± 0.13^c^	5.22 ± 0.14^c^	5.38 ± 0.15^c^
Water ultrasonic	2.88 ± 0.06^cd^	3.02 ± 0.06^bc^	4.18 ± 0.09^bc^	4.72 ± 0.1^c^	5.27 ± 0.11^c^	5.43 ± 0.11^c^
Ethanol:water(50:50)ultrasonic	3.1 ± 0.05^b^	3.25 ± 0.06^a^	4.5 ± 0.08^a^	5.08 ± 0.09^a^	5.66 ± 0.09^a^	5.84 ± 0.1^a^
Ethanol ultrasonic	2.89 ± 0.06^cd^	3.03 ± 0.07^bc^	4.2 ± 0.09^bc^	4.74 ± 0.1^bc^	5.29 ± 0.11^bc^	5.45 ± 0.12^bc^
BHA	5.11 ± 0.16^a^					

Note. Different letters in the column indicate significant differences (*p* < 0.05).

### 3.9 Antibacterial activity of *Arum maculatum* extracts by microdilution method

The minimal inhibitory concentration (MIC) and minimal bactericidal concentration (MBC) of *Arum maculatum* extracts were determined against some food‐borne pathogens. Various bacterial strains showed different sensitivity to these extracts. It seems that all extracts were more effective against Gram‐positive than Gram‐negative bacteria and the most sensitive microorganisms in this test were *S. aureus* and *L. monocytogenes* as Gram‐positive bacteria with the lowest MIC value 12.5 mg/ml (Table 5). While different plant extracts in previous studies indicated various antimicrobial potential against these bacteria. For example, Oliveira et al. (2008) reported considerable lower MIC value (0.1 mg/ml) for walnut green husks aqueous extracts against *S. aureus* and Shen et al. (2014) illustrated higher MIC (300 mg/ml) for blueberry extracts against *L. monocytogenes*. Moreover, *Arum maculatum* essential oil demonstrated higher MIC against *S. aureus* (32 mg/ml) than our study, while it had greater effect on *E. coli* (MIC *=* 4 mg/ml) (Kianinia & Farjam, 2018). Also, other plant extracts have shown similar trends for inhibition of bacterial growth in the study by Negi and Jayaprakasha (2003). The MIC values variations between Gram‐negative and Gram‐positive bacteria may reflect differences in their cell surface structures. In fact, Gram‐negative bacteria have a lipopolysaccharide outer membrane and the transfer of molecules is achieved through the cell membrane. The antimicrobial effect is related to the ability of the compounds based on shape and size to penetrate into the outer membrane and reach their site of action (Kavak, Altıok, Bayraktar, & Ülkü, 2010). Moreover, less sensitivity of Gram‐negative bacteria to extracts and essential oils is due to the hydrophilic cell wall of Gram‐negative bacteria that it prevents penetration of hydrophobic oil and the accumulation of substances in the bacterial cell membrane (Sumbul, Ahmad, Asif, & Akhtar, 2011). Burt (2004) also reported that the effects of different plant extracts such as lavender, rosemary, cinnamon, and oregano on Gram‐negative bacteria were lower than Gram‐positive bacteria. Our results also agree with Behdani, Ghazvini, Mohammadzadeh, and Sadeghian (2009) that demonstrated aqueous extract of henna has little antimicrobial activity against Gram‐negative bacteria such as *Pseudomonas aeruginosa* and *Escherichia coli*, but has excellent antimicrobial activity against Gram‐positive bacteria such as *Staphylococcus aureus*. The result of microdilution assay showed that in many cases the *Arum maculatum* extracts possesses bacteriostatic properties in lower concentrations and bactericidal properties in the higher concentrations (Table 5). The same results were observed by Simões‐Gurgel et al. (2012) on the antibacterial activity of methanol extract of *Cleome rosea* vahl.

**Table 5:** Assessment of MICs and MBCs for *Arum maculatum* extracts in microdilution assay

Bacteria	Extracts											
	WE‐M		WEE‐M		EE‐M		WE‐US		WEE‐US		EE‐US	
	MIC	MBC	MIC	MBC	MIC	MBC	MIC	MBC	MIC	MBC	MIC	MBC
*E. coli*	100	100	25	50	50	100	50	100	50	50	25	50
*S. aureus*	50	100	50	50	50	50	25	50	25	50	12.5	50
*L. monocytogenes*	50	50	50	50	50	50	50	100	25	50	12.5	100
*S. enteritidis*	50	50	50	100	100	100	50	100	50	50	25	100
*P. aeruginosa*	100	100	100	100	100	100	50	50	50	100	50	100

Notes. Values are based on mg/ml of the extracts.

WE: water extract; EE: ethanol extract; WEE: water‐ethanol extract (50:50).

M: maceration extraction, US: ultrasonic extraction.

The result of microdilution assay showed that in many cases the *Arum maculatum* extracts possess bacteriostatic properties in lower concentrations and bactericidal properties in the higher concentrations (Table 5). Among different extracts, ultrasonic extracts were the most effective against all pathogen bacteria in this study so that the lowest MIC and MBC associated with EE‐US and WEE‐US, respectively.

It can be related to the presence of more active compounds in this extract than other *Arum maculatum* extracts. According to Table 1, ultrasonic extracts had a higher amount of phenolic, tannins, flavonoids, and tocopherols than maceration extracts. In many types of research, natural phenolic compounds of different plants have shown potential antiviral, antibacterial, and antiparasitic effects (Nitiema et al., 2012; Rauha et al., 2000; Zandi et al., 2011).

Shahidi and Naczk (1995) reported that an antimicrobial action of phenolic compounds was related to the inactivation of cellular enzymes, which depended on the rate of penetration of the substance into the cell or caused by membrane permeability changes. All our data suggested that the antimicrobial efficacy of ultrasonic extracts was associated with their specific phenolic composition.

### 3.10 Antimicrobial activity of *Arum maculatum* extracts by agar diffusion methods

**Figure 1:**
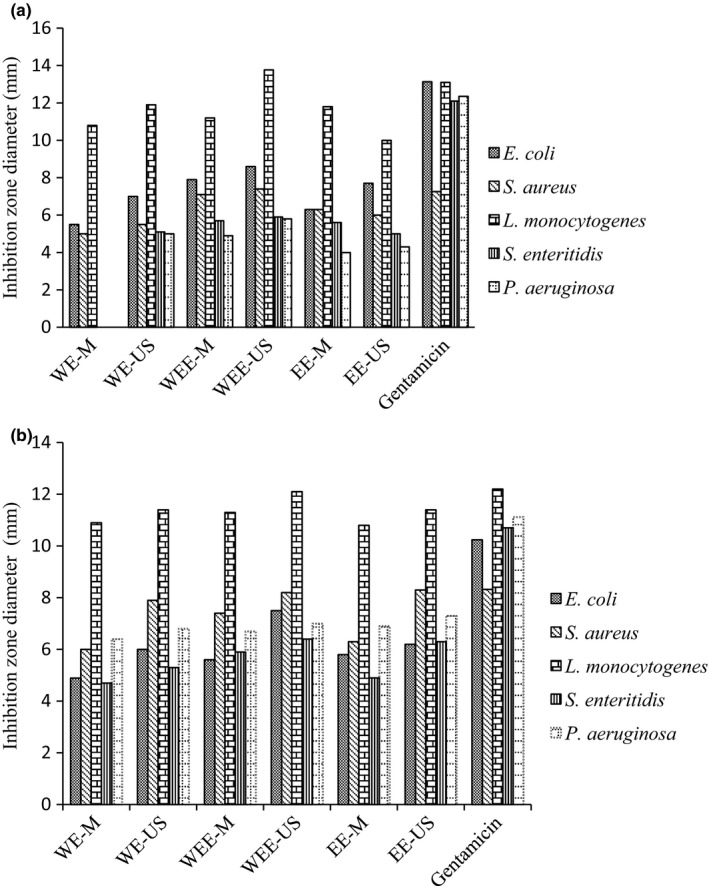
Antimicrobial activity of Arum maculatum extracts using (a) disk diffusion assay, (b) well diffusion assay

The antimicrobial activities of all *Arum maculatum* extracts were examined in the disk diffusion and well diffusion assays against five bacteria. The data were defined as the diameter of inhibition zone (mm). The variable results of two tests are illustrated in Figure 1. Generally, the results of the disk and well diffusion assays for all extracts were relatively similar, and *L. monocytogenes* as Gram‐positive bacteria indicated the largest zone of growth inhibition (13.77 mm) compared with others. Moreover, Gram‐negative tested bacteria were detected as resistant strains, and all *Arum maculatum* extracts showed low antimicrobial properties against *P. aeruginosa* and *S. enteritidis* in the disk and well diffusion methods, respectively (Figure 1a,b). It does not mean that Gram‐positive bacteria are always more vulnerable (Burt et al., 2007). For example, there was an exception in disk diffusion assay and *E. coli* appeared higher inhibition zone than *S. aureus* (Figure 1). Extract solvent (water) was used as a control and has shown no antimicrobial activity in all diffusion method. Furthermore, growth of *S. enteritidis* and *P. aeruginosa* was not inhibited by water extract of maceration method in disk diffusion assay. On the other hand, all extracts from ultrasonic created bigger inhibition zone than extracts from maceration. In two diffusion assays, gentamicin antibiotic disk (10 micrograms per disc) was used as a positive control or standard and results showed that the *Arum maculatum* extracts inhibitory effect on all bacteria was less than gentamicin except *L. monocytogenes*. These findings were in accordance with the results of Khosravinia, Ziaratnia, Bagheri, and Marashi (2013) that surveyed the antimicrobial activity of cell extracts derived from in vitro culture of Caraway (*Bunium persicum*).

Generally, the strength of the antimicrobial substance in agar diffusion methods is determined by measuring the diameter in comparison with a standard. In theory, there is a linear relationship between the size of the halo and the logarithm of the active ingredient concentration in plant extracts. Also, the inhibition area depends on the ability of the antibacterial compounds to diffuse uniformly through the agar. This phenomenon was noted in many reports (Rauha et al., 2000).

As a result, in all assays, the bactericidal effect depends on the concentration of active components in extracts and type of bacterial strains. The water‐ethanol extracts (50:50) were strongly bactericidal than others and provide larger inhibition zone (mm) for all tested bacteria, especially *L. monocytogenes*. Since the solubility of antimicrobial compounds in ethanol:water (50:50) may be better than other solvents. In fact, the polarity of ethanol–water solvent is high and phenolic compounds with polar nature dissolve in it well. The results of this study were different from other works by using other plant extracts such as Negi and Jayaprakasha (2003). Various factors including the type of plant, solvent, plant tissue, time and place of plant collection, climate change and geographical conditions of the region, conditions and facilities of laboratory, etc., can be the cause of these difference (Khosravinia et al., 2013).

## 4 CONCLUSIONS

In this paper, antioxidant and antimicrobial activities of a specie of Iranian *Arum maculatum* leaves extracts were investigated, which have rarely been reviewed. The influence of the solvent (ethanol, water, and ethanol:water (50:50)) and extraction method (maceration and ultrasonic) on different properties of extracts were demonstrated. The highest extraction yield was obtained with ethanol:water (50:50) solvent in ultrasonic method. Also ultrasonic waves could significantly improve total phenolic, tannin, flavonoid, and tocopherol content of the produced extracts. Our results also indicated a high correlation between total phenolic content and antioxidant properties. These findings confirmed the better antioxidant activity of ultrasonic extracts than maceration extracts. Antioxidant activity was measured by three methods (DPPH radical‐scavenging activity, OSI (Rancimat test), and beta‐carotene/linoleic bleaching inhibition). In addition, all *Arum maculatum* extracts showed antibacterial potential against some Gram‐positive and negative food‐borne bacteria by using agar diffusion as well as microdilution method and *Listeria monocytogenes* was identified as the most sensitive bacterium.

Generally, our results showed that Iranian *Arum maculatum* can be used as an easily accessible source of natural bioactive compounds with antioxidant and antibacterial properties that can be a proper substitute for synthetic antioxidants and artificial antimicrobials to use in various food products. Also, the potential showed by this plant can lead to the valorization of a by‐product that nowadays has a scarce use.

On the other hand, further studies are needed to isolate and characterize the bioactive compounds that responsible for the bioactive activity was observed here. In continuation, more researches should be performed in order to identify the chemical structure of main antioxidants in *Arum maculatum*. At last, to better manage consequences arising from oxidative damage, detailed information on the structure of the most active compounds of the plant must be investigated and other further biological tests should be conducted.

## CONFLICT OF INTEREST

The authors declare no conflict of interest.

## ETHICAL STATEMENT

This study does not involve any human or animal testing.
